# Biomaterials and Clinical Application of Dental Implants in Relation to Bone Density—A Narrative Review

**DOI:** 10.3390/jcm12216924

**Published:** 2023-11-03

**Authors:** Angkoon Khaohoen, Tanapon Sornsuwan, Pisaisit Chaijareenont, Pongsakorn Poovarodom, Chaiy Rungsiyakull, Pimduen Rungsiyakull

**Affiliations:** 1 Department of Prosthodontics, Faculty of Dentistry, Chiang Mai University, Chiang Mai 50200, Thailand; angkoon_khaohoen@cmu.ac.th (A.K.); pisaisit.c@cmu.ac.th (P.C.); pongsakorn_poova@cmu.ac.th (P.P.); 2 Department of Restorative Dentistry, Faculty of Dentistry, Naresuan University, Phitsanulok 65000, Thailand; tanapons@nu.ac.th; 3 Department of Mechanical Engineering, Faculty of Engineering, Chiang Mai University, Chiang Mai 50200, Thailand; chaiy.rungsiyakull@cmu.ac.th

**Keywords:** dental implants, titanium, zirconia, biomaterial, bone density, marginal bone loss, osseointegration

## Abstract

Titanium has been the material of choice for dental implant fixtures due to its exceptional qualities, such as its excellent balance of rigidity and stiffness. Since zirconia is a soft-tissue-friendly material and caters to esthetic demands, it is an alternative to titanium for use in implants. Nevertheless, bone density plays a vital role in determining the material and design of implants. Compromised bone density leads to both early and late implant failures due to a lack of implant stability. Therefore, this narrative review aims to investigate the influence of implant material/design and surgical technique on bone density from both biomechanical and biological standpoints. Relevant articles were included for analysis. Dental implant materials can be fabricated from titanium, zirconia, and PEEK. In terms of mechanical and biological aspects, titanium is still the gold standard for dental implant materials. Additionally, the macro- and microgeometry of dental implants play a role in determining and planning the appropriate treatment because it can enhance the mechanical stress transmitted to the bone tissue. Under low-density conditions, a conical titanium implant design, longer length, large diameter, reverse buttress with self-tapping, small thread pitch, and deep thread depth are recommended. Implant material, implant design, surgical techniques, and bone density are pivotal factors affecting the success rates of dental implant placement in low-density bone. Further study is required to find the optimal implant material for a clinical setting’s bone state.

## 1. Introduction

Dental implants generally represent the go-to procedure for replacing missing teeth because of their high success rate and attractive results. However, compromised bone quality leads to both early and late implant failures in 7.7% of cases [1]. According to this result, achieving initial stability in low-density bone, which is essential for osseointegration, is a challenging task. With the advancement of technology, numerous studies strive to enhance fixture designs and surgical techniques based on soft bone [2,3,4]. The factors that can affect the success rate of dental implants include implant material, implant design, surgical technique, patient health, or host bed [5].

Dental implant materials and designs (length, diameter, and macro- and microgeometry) affect how much stress and strain develops around the peri-implant tissue, which indicates the chance of successful osseointegration [5,6]. Consequently, the incompatibility between the elastic modulus of the implant material and bone may cause the “stress-shielding” phenomenon, which is related to peri-implant bone resorption [7,8,9]. In other words, the closer the elastic modulus implants are to the bone tissue, the better the distribution of tension. To lessen this difference, numerous studies have suggested developing either porous implants that serve as a scaffold for internal bone growth and lower the modulus of elasticity or innovative materials alloys with lower moduli [10,11,12].

Titanium has been utilized in the fabrication of dental implants since approximately 1981 [13]. According to the osseointegration concept, Brånemark first demonstrated how titanium implants induce bone integration and discovered that the titanium oxide (TiO_2_) layer might be responsible for establishing direct bone–implant contact [14]. Due to the TiO_2_ passivation coating that covers the metal surface, titanium demonstrates a strong corrosion resistance [15] in addition to desired mechanical properties, such as fatigue strength and wear resistance. Titanium and its alloys have an advantage in biological reactions due to their complete inertness in the body environment and biocompatibility [16]. Zirconia may be an alternative to titanium for individuals worried about a metallic look in the esthetic zone or metal allergies [17]. Currently, commercially available zirconia implant fixtures involve 3 mol% yttria–tetragonal zirconia polycrystal (3Y-TZP), ceria-stabilized zirconia/alumina nanocomposite (Ce-TZP/Al_2_O_3_ or NanoZR), and alumina-toughened zirconia (ATZ) [18]. Even though several studies have shown that zirconia implants have an osseointegration capacity comparable to that of titanium implants [19,20], long-term clinical data regarding mechanical [21] and biological aspects are still required.

Macrogeometry has a remarkable impact on implant stability. In cases of low-density bone, it has been proved that a conical shape achieves better initial fixation than a cylindrical design. Additionally, implant microdesign—which includes thread shape, depth, pitch, and width—is crucial for both mechanical and biological responses [22].

An additional key indicator of an implant’s success rate is the appropriate surgical technique. Various approaches are suggested by several studies to prevent failure mode, particularly in low-density bone. For instance, osseodensification is a strategy that increases drill torque without damaging biological tissue from excessively high temperatures. Under-drilling is an adaptive technique that skips the final drill protocol to enhance implant primary stability and interfacial contact. Still, the drilling guideline is debatable and frequently based on clinically perceived sensibility [2,23,24].

To reduce the high risk of implant failure associated with low bone density, researchers attempt to develop appropriate implant designs and surgical techniques to achieve the optimization guidelines for each bone condition. Nevertheless, there have been a limited number of studies regarding implant material, implant design, and surgical technique in relation to bone densities. Therefore, the purpose of this narrative review is to investigate the influence of implant material, implant design, and surgical technique on bone density from both biomechanical and biological standpoints. An additional aim is to identify the association between implant material and bone density in terms of clinical outcome.

## 2. Materials and Methods

The focused (PICO) question to be addressed by this review is as follows: “How do dental implant material, implant design, and surgical technique affect bone density in terms of biomechanical and biological aspects?”

P: Edentulous area treated with implant surgery.

I: Dental implant materials (titanium, zirconia, other materials).

C: Different bone densities of hard bone D1, medium bone D2–D3, soft bone D4, and cortical bone D123, no cortical D4. In this study, we classified bone densities according to high (D1–D2) and low (D3–D4).

O: Stress concentration, bone–implant interface contact (BIC), push-in values, removal torque, fracture resistance, low-temperature degradation (LTD), mean bone loss (MBL), and success and survival rates.

S: Randomized controlled or non-randomized controlled trials, case reports, animal studies, in vitro studies, finite element analysis, and review articles.

An initial electronic search of PubMed/Medline, Scopus, Embase, and other gray sources was carried out for publication dates up to 2023 with a limit of 10 years and English language search criteria. The search terms were applied in Table 1.

**Figure 1:**
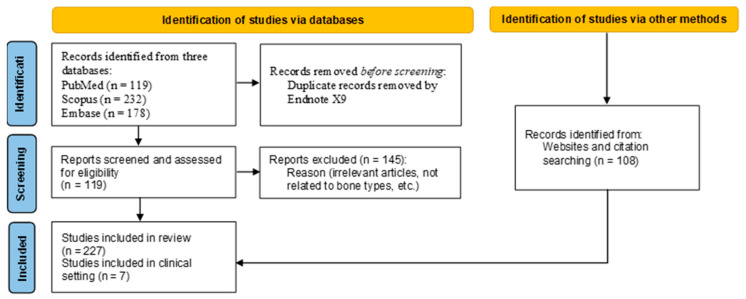
Flow diagram of search procedure for this narrative review.

Included articles were those that investigated the biomaterial of dental implants related to bone mineralized density in terms of biomechanical, biological, and clinical outcomes. After preliminary searching, 529 articles were found. Duplicated articles were eliminated, and irrelevant articles were filtered out due to ineligibility based on the criteria. A total of 119 articles from the findings were included for consideration in this review. Gray literature was also taken into consideration for this analysis. For bone density in the clinical setting, 7 included articles were examined (Figure 1). Software for managing references (EndNote version 9) was employed.

After evaluating the relevant articles, the included studies were recorded on an Excel spreadsheet (Microsoft Corporation, Redmond, WA, USA). The data were grouped depending on the study design, bone types/density, dental material types, and outcome measurements such as mean bone loss, success rate, and survival rate. 

## 3. Results

### 3.1. Bone Response to Dental Implant Materials

Both biomechanical and biological events can be achieved by osseointegration, referring to a direct bone–implant interface without the interposition of non-bone tissue—a higher degree of osseointegration results in the increased function and primary stability of dental implants [25]. Biomechanical factors are related to the clinical bone response to surgically placed dental implants at the time of insertion. Related factors such as surgical technique, bone density, and the utilization of adjacent graft material and device capabilities are crucial to encouraging and achieving initial bone growth. This initial stability must persist throughout the demineralization phase of bone injury. At 5 or 6 weeks, basic multicellular unit (BMU) remodeling begins, replacing the woven bone with more dense lamellar bone. The bone mineral density around the implant body was then increased for up to two years [26,27].

As well as the implant-related factor and prosthetic relevance (prosthetic design, force magnitude and direction, and load distribution) [28], one of the critical parameters for achieving both osseointegration and mechanical stability is the compatibility between the bone quality of the patient and the stiffness of the implant [9]. As a result, to optimize implant design based on the patient’s specific bone quality, the properties of the dental implant should be correspondingly adjusted.

#### 3.1.1. Bone Density

Bone density, often known as bone quality, refers to the internal structure of bone [29]. Depending on the mechanical properties of the bone, bone quality can be divided into two aspects: density and modulus of elasticity [30]. Regarding bone structure, cortical bone is a crucial feature affecting the primary stability of implant placement, whereas cancellous bone is related to blood supply [31]. According to Misch’s classification [32], bone is categorized into the four following types according to the mineralized density and cortical thickness:

D1 bone is primarily dense cortical bone.

D2 bone comprises dense to thick, porous cortical bone with coarse trabecular bone underneath.

D3 bone is composed of thinner porous cortical bone and fine trabecular bone.

D4 bone comprises only fine trabecular bone.

Generally, the bone quality depends upon the jaw location, showing that the mandible surrounding the implant is superior in quality to the maxilla (Table 2). Significant differences exist between healthy and medically compromised subjects, age, and gender. For instance, Young’s modulus of bone can drop by up to 40 to 50% in an osteoporotic patient compared to a normal patient [33]. Regarding gender, males have higher mean bone density values at implant sites than females because of hormonal differences. Furthermore, older females have been found to have lower bone mineral densities than older males [34].

Several studies have classified bone density before implant placement based on Hounsfield units (HUs) in computer tomography (CT) examinations. Hounsfield scale is a semiquantitative technique for determining X-ray attenuation. In the HU scale, distilled water is arbitrarily assigned a value of 0 HU and air defined as −1000 HU (black on the gray scale), and characterized bone quality is represented by a value of +150 to +350 (D_4_), +350 to +850 (D_3_), +850 to +1250 (D_2_), and >1250 (D_1_) [35], as shown in Table 3. Due to its high resolution and minimized radiation exposure compared to traditional CT, cone beam computed tomography (CBCT) is one of the imaging modalities used to measure bone density. However, the gray values (GVs) from CBCT cannot be directly translated to HUs due to the lack of clinical research, which is needed to justify its use as a diagnostic tool [36,37].

#### 3.1.2. Bone Remodeling and Bone Density

##### Peri-Implant Bone Strain

Mechanical loads result in bone apposition and deformation or strain, which preserve bone mass and structure through remodeling and modeling processes. Frost’s theory proposes that strain magnitude is the stimulus for bone functional adaptation. Regarding loading, the term “strain” describes the relative alteration in bone length or the deformation of bone tissue. Bone resorption occurs when the strains are between 50 and 100 microstrains. In contrast, pathologic bone occurs as microdamage or fracture if the strains have limited bone capacity or greater than 25,000 microstrains. There is a short window, between 1500 and 3000 microstrains, where non-physiologic or mild overloading causes bone gain [41,42].

##### Stress Distribution in Peri-Implant Bone

The density of each bone varies in strength and impacts elastic modulus, which influences the distribution of stress and strain at the bone–implant contact and the BIC (bone–implant contact) percentage. Low cancellous bone density increases bone strains while decreasing implant stability, which may result in implant failure. In addition, low bone density can also make it more challenging to place the implant in the desired location and angle. The initial bone density provides mechanical immobilization during healing and better stress transmission from the prosthesis to the bone–implant interface [43]. Consistent with previous studies [44,45], higher stress levels were observed in D4-type bone compared with the other bone types. The stress was generally concentrated in the cervical part of the implant socket. In other words, decreased bone density and cortical bone thickness increase the stress on the bone and implants, which can result in bone resorption and implant failure. On the contrary, higher bone density can provide better implant stability and a more evenly distributed occlusal load, reducing the stress and strain on the implant and the surrounding bone tissue [46].

In terms of mechanical incompatibility, differences in the elastic modulus of bone tissue and implant materials (Table 4) induce “stress shielding” by absorbing tension and transferring less stress and deformation to the bone tissue, leading to peri-implant bone loss and aseptic implant loosening [47]. In other words, the closer the elastic modulus implants are to the bone tissue, the better the distribution of tension. However, finite element analysis shows that using softer materials with similar strength to the bone provides no benefits in better stress distribution to the peri-implant bone. For example, polyetheretherketone (PEEK) material does not affect the yielding or fracture of implant components; using PEEK as an implant or abutment results in more significant deformation than titanium under static load [48]. More data from controlled clinical trials on PEEK implants are needed. Titanium alloy or zirconia implants currently remain the materials of choice due to their excellent mechanical qualities, such as stiffness. In addition, the use of advanced materials, such as nanostructured or porous materials and novel alloys, can provide a better stress distribution by reducing its elastic modulus and improving the implant’s biological response in subsequent osseointegration, particularly under compromised bone conditions. Considerably, implant design, implant location, manufacturing method, and functional graduation are also essential factors to consider in addition to implant material [12,46].

Based on a thorough assessment of the patient’s bone quality, the mechanical response of different implant designs and materials in different bone densities should be demonstrated as valuable information for clinical decision-making.

##### Clinical Assessment Tool

Insertion torque (IT) and resonance frequency analysis (RFA) are clinical measurements of implant stability. The insertion torque value (ITV) represents the resistance to a rotational load against the implant to the bone during implant insertion measured in Ncm [50]. According to Aparicio and colleagues, 30 to 50 Ncm provides acceptable implant stability. Some clinicians claim that a high ITV can act as a stimulus for faster osseointegration. A systematic review stated that a high ITV is required for immediate or early loading [51].

ITV, however, is largely dependent on the area of contact with bone, notably the cortical layer [52]. Moreover, the implant-cutting design capacity and bone friction are crucial determinants of distinct bone effects, resulting in high or low ITV [53]. A retrospective study [54] evaluating the mechanical effect of a drilling technique based on bone quality discovered that the bone type and implant diameter significantly influenced ITV. Compared to bone types 3 and 4, type 1 demonstrated a higher ITV. Even if the dense bone type achieves a high ITV value, the marginal bone may lose mean bone mass due to excessive stress and strain. Therefore, before implant placement in dense bone, the operator should minimize ITV to reduce the local pressure using taps or countersinking for the cortical layer.

RFA is a different technique that works by sending a frequency signal to a screwed transducer, which causes the implant to vibrate. The RFA output is expressed as an implant stability quotient (ISQ). The function of the stiffness of the bone-to-implant contact during treatment and follow-up is indicated by the greater ISQ value [55]. Several studies confirm that ISQ values below 70 and 75 at implant placement or after 3–4 months of healing indicate implants with a higher risk of failure [24,56].

Removal torque (RTQ) is a destructive test determining the critical torque value for disrupting the bone-to-implant interlocking by unscrewing the implant. This test, unutilized in clinical research, can be used to assess the quality of osseointegration in terms of bone-to-implant contact. Like the ITV, the RTQ is influenced by bone quality, implant geometry, and implant stability [57,58].

##### Histological Assessment Tool

The higher ISQ value indicates the function of the stiffness of the BIC. The BIC ratio is correlated with the biomechanical properties of the bone-to-implant interface and increases during bone healing [59]. Histological analysis of bone-to-implant interfaces has determined the presence of mineralized tissue in contact with the implant surface rather than fibrous tissue. Nonetheless, it is still being determined how the BIC % can be translated into osseointegration [60]. According to Albrektsson and coworkers [61], good integration accounts for 60% of BIC. Different results can be obtained depending on the bone type, healing time, and implant type.

### 3.2. Material Used for Dental Implants and Their Properties

Titanium has recently become the material of choice for dental implant fixtures due to its exceptional qualities and excellent balance of rigidity and stiffness. Since zirconia is a soft-tissue-friendly material and caters to aesthetic demands, it is an alternative material to titanium implants. Additionally, PEEK is a polymer material with excellent mechanical properties and superior biocompatibility because it has a low Young’s modulus comparable to surrounding bone, which influences an optimal load transfer [62].

#### 3.2.1. Biomaterial of Dental Implants

##### Metal and Metal Alloy

Titanium and Ti-6Al-4V

Titanium is a frequently used material for dental implants, mainly because of its biocompatibility and capacity to promote osseointegration. The ability of titanium metal to react with air and generate hydroxyl and hydroxide groups endows it with a high capacity for resisting corrosion. This reaction results in the formation of titanium dioxide, which is the most reported in dental implant fields [63]. Currently, there are four grades of commercially pure titanium (CpTi), ranging in purity from 98.0% to 99.6% and processing oxygen content, as well as two different types of titanium alloys made of Ti-6Al-4V and Ti-6Al-4V Extra-Low Interstitial alloys in mechanical properties, such as strength, ductility, and corrosion resistance [64].

Grade IV CpTi is the most commonly utilized variety due to its high oxygen content (0.4%) and, thus, excellent mechanical strength. The alloy Ti-6Al-4V, often known as grade V titanium, is widely used in orthopedics because of its superior strength and lower Young’s modulus [13]. However, aluminum (Al) and vanadium (V) may affect bone mineralization and type IV allergic reactions, respectively [65]. To avoid an adverse biological response, vanadium-free alloys and non-toxic components such as Nb, Ta, Zr, and Pd are being developed [64].

Titanium and Titanium–Zirconium Alloy

A novel alloy (Roxolid^®^, Straumann, Basel, Switzerland: TiZr1317) has been produced based on the binary formation of 83–87% titanium and 13–17% zirconium [66]. It outperforms CpTi and Ti-6Al-4V in terms of tensile (953 MPa) and fatigue strength (230 N) [67]. Furthermore, this alloy material exhibits good biocompatibility as pure titanium [68].

Titanium Alloys in 3D Printing

In recent years, 3D printing technologies, also known as additive manufacturing (AM) technologies, have been successfully applied in implant dentistry because they are the alternative method for generating implant products. Moreover, they have allowed for the fabrication of custom implants with microscale resolution. Metallic implants were frequently created using selective laser fusion (SLM) and electron beam fusion (EBM) procedures [69].

##### Ceramics

For people concerned about a metallic appearance in the esthetic zone or metal allergies, ceramics are an alternative material to titanium [4]. Ceramics are known as inert materials and have good physical properties. They are widely used as a coating material for metal implants [70] and a substrate for fabricating dental implants [71]. Presently, commercially available zirconia implant fixtures involve Y-TZP and ATZ. Details on the manufacturer, brand name, material, and design of the zirconia implant system are given by Ban [18].

Yttria–Tetragonal Zirconia Polycrystal (Y-TZP)

Zirconium dioxide (ZrO_2_), often known as zirconia, is a polymorphic material occurring in three temperature-dependent forms: monoclinic (stable from room temperature to 1170 °C), tetragonal (from 1170 to 2370 °C), and cubic (from 2370 °C to the melting point, 2680 °C). When cooling, there is a significant alteration in zirconia unit cell volume, resulting in structural defects that affect the mechanical properties. Because of the spontaneous phase shift of zirconia from tetragonal to monoclinic, doping agents (CaO, MgO, Y2O_3_) are used to stabilize the structure to create partially stabilized zirconia (PSZ) or tetragonal zirconia polycrystal (TZP) [72,73]. The microstructure of 3Y-TZP ceramics for dental applications comprises 3 mol% yttria as a stabilizer and up to 98% small equiaxed tetragonal grains, occasionally with a small amount of cubic phase. The mechanical characteristics of 3Y-TZP depend on the grain size, determined by the sintering temperature [74,75]. Recently, Y-TZP used as an oral implant material has demonstrated exceptionally high strength (>1200 MPa) [76]. Nevertheless, low-temperature degradation or aging can reduce the fracture resistance of the material [77] and generate microcracks or micro-roughness on the surface caused by a spontaneous and progressively change in Zr_2_O grains from the tetragonal to the monoclinic phase in the presence of water molecules. Then, moisture can seep deeper into the material, accelerating the aging phenomenon [78]. A novel ceria-stabilized zirconia-based composite was developed to reduce the aging in dental ceramics [79] that is not prone to aging due to the stabilization using cerium rather than yttrium [80]. According to an in vitro investigation, this innovative zirconia-based composite is appropriate for reduced-diameter one-piece design implants in the anterior jaw regions [81].

Alumina-Toughened Zirconia (ATZ)

ATZ, a zirconia–alumina composite, is a composite ceramic material of 20 vol% alumina and 80 vol% zirconia with 3 mol% yttria. The addition of alumina significantly improves flexural strength, fracture toughness, and resistance of the material to surface degradation [82]

##### Polyetheretherketone (PEEK)

PEEK has been used as an alternative to metals for implants since 1998. It has emerged as an option for individuals desiring metal-free restorations in cases of bruxism and allergic responses. Due to its stiff semicrystalline nature, hardness resembling bone, excellent mechanical capabilities, and superior biocompatibility, PEEK is employed as a biomaterial for implant rehabilitation [62].

PEEK material has a Young’s modulus of 3.6 GPa in pure form, 18 GPa in carbon-reinforced PEEK (CFR-PEEK), and 12 GPa in glass fiber-reinforced PEEK (GFR-PEEK) [49]. Because PEEK has Young’s modulus close to that of bone, it allows for better dispensation of masticatory force around the implant and exhibits less stress shielding than titanium implants [83].

Most polymers, such as PEEK, have low surface energy, making them bioinert, and do not provide osteoconductive properties. As a result, PEEK stimulates less osteoblast differentiation than titanium [62]. To induce more bone formation on the PEEK surface, various modifications such as hydroxyapatite (HA)-covered PEEK, TiO_2_ coating on PEEK, or sandblasting (nFA/PEEK) can improve the PEEK surface by rendering it more biologically compatible [84].

Furthermore, PEEK influences biofilm structure and reduces the potential of peri-implant inflammation. However, because there have been very few clinical trials on PEEK as a dental implant, conclusive evidence is awaited on whether it can truly replace titanium as a dental implant [62].

#### 3.2.2. Functional Properties of Dental Implant Materials to Bone Density

The mechanical properties of implant materials, such as stiffness, strength, ductility, and toughness, describe their capacity to withstand forces and displacements measured by uniaxial tensile tests [85]. Stress, which defines the force applied to a material, is classified into three types: tensile, compressive, and shear.

Strength is generally defined as the ability of the prosthesis to withstand applied stress without fracture (ultimate strength) or permanent deformation (yield strength). As a property, strength is not as reliable for estimating the survival probabilities of brittle material prostheses over time compared to fracture toughness, which more clearly describes the resistance to crack propagation of brittle materials [86]. Flexural strength, also called bending strength or modulus of rupture, is a strength test of the material’s ability to sustain bending forces applied perpendicular to its longitudinal axis [87].

Elastic modulus, measured by the ratio of elastic stress to elastic strain, is a term used to describe a material’s relative stiffness or rigidity. This property impacts the strength and fatigue strength of the materials as well as the functionality of manufactured implants [47].

Ductility represents the ability of a material to resist a sizeable permanent deformation under a tensile load up to the point of fracture. For example, material A is the most ductile, as shown by the most extended plastic strain range. In contrast, material B is brittle, defined as having no plastic deformation and breaking at the proportional limit [86].

The overall area between the elastic and plastic zones, from zero stress to fracture stress, is related to toughness. Fracture toughness measures the energy required to propagate critical flaws in the structure. For instance, a tough material is generally strong, whereas a strong material is not necessarily tough [86].

##### Elastic Modulus, Stiffness

After an implant completes osseointegration, the chewing stress is transferred to the bone tissue surrounding the implant body. As a result of the mismatch between the stiffness of the implant material and the peri-implant bone, the stress imbalance can lead to marginal bone loss and implant failure [88]. This stress-induced marginal bone destruction can be described using the “composite beam analysis” principle. In the case of two materials with different elastic moduli, the primary point of contact between the two materials is where the greatest stress is concentrated [89]. According to Hooke’s law, material stiffness depends on its modulus. In the case of the bone–implant system, the bone tends to create more significant deformations. Thus, controlling the variables determining how to reduce the transferred stress is critical, including load type, implant treatment protocol, design component, and peri-implant bone quality [90]. Ti-6Al-4V alloy is the most frequently utilized in the fabrication of dental implants because of its superior elastic modulus and tensile strength [91]. In cases of limited bone availability, a rigid alloy called “Ti-Zr” has been developed [92]. Zirconia implants, such as Y-TZP, have become more popular as aesthetic concerns have increased. To improve the elastic properties to more closely resemble the characteristics of pristine bone with better biomimetics and biocompatibility, many researchers have attempted to develop novel alloys [93] or improve the implant surface characteristics (porosity) [94,95]. Therefore, by reducing the stiffness of the implant materials, many studies have attempted to determine the optimal mechanical characteristics of titanium-based materials. To modify its mechanical properties, alloy elements are often added, including Nb, Zr, Ta, and Sn. Furthermore, producing a porous structure employing 3D printing technology lessens implant stiffness, improves stress transmission, and accelerates the formation of new bone and osseointegration [47].

PEEK is widely used to replace titanium or zirconia implant materials because carbon fiber can be added to it to produce carbon fiber PEEK (CFR-PEEK) with varying elastic moduli, which are within the range of the bones’ elastic modulus [96,97]. Interestingly, 30% carbon fibers PEEK (30CFR-PEEK) shows higher stress concentration and deformation at the bone–implant interface, even though reinforcing PEEK with carbon and glass fiber—by determining the optimum quantity, size, and shape of carbon fibers—is the most effective way to improve mechanical properties and prevent stress shielding. However, these drawbacks can be lessened using 60% carbon fibers PEEK (60CFR-PEEK) [98]. In clinical settings, PEEK is expected to replace titanium and zirconia implant materials due to its low stress shielding. Furthermore, PEEK has greater application benefits for patients with poor bone conditions, bruxism, and esthetic concerns [99]. The functional properties of various implant materials are presented in Table 5.

The implant geometry significantly affects the implant treatment because it can enhance the mechanical stress transmitted to the bone tissue, causing marginal bone loss [100]. To be more precise, unbalanced load distribution can be improved by modifying implant macro- or microgeometries. According to one literature review, a hybrid design, which combines conical and cylindrical forms, has the highest primary stability because it can distribute stresses more uniformly and incorporate more bone. A conical design, which is wider at the base and narrower at the top, also provides better initial stability and load distribution. This result can reduce marginal bone loss when compared to a cylindrical form [101]. In cases requiring immediate implant insertion, conical implant systems with double threads and a low thread helix angle should be used [102]. In addition to implant shape, a previous study concluded that primary stability significantly increases with implant length. Longer implants can affix to more bone in soft bone situations because they have a greater surface area for osseointegration, increasing primary stability. Nevertheless, different implant lengths and diameters do not appreciably alter the primary stability properties in environments of dense bone [103]. Diameter becomes a more decisive factor as soon as implant length is sufficient since the stress occurs at the implant neck, where bone loss initiates at an early implantation stage. In soft bone, wider-diameter implants improve primary stability and functional surface area, allowing even load distribution [104].

The implant’s thread design is crucial for its early mechanical primary and ensuing secondary biological stability [105]. Dental implants with a narrow pitch have an increased implant surface and more threads per implant length, which might improve load distribution [106].

The higher the thread depth, the more the surface and the load distribution increase. Greater thread depths may benefit the more excellent functional surface, particularly with softer bone and high occlusal stresses [107], while axial forces distributed as compressive forces with square and buttress threads can be transformed into shear and compressive forces using V-shaped reverse buttress threads [108]. Thread designs with a depth of 0.34 to 0.5 mm and a width of 0.18 to 0.3 mm are reportedly advantageous [108]. Moreover, the thread’s face angle directly controls how much force the implant applies to the surrounding bone [109]. The optimal pitch, with more than 0.8 mm and greater thread-to-thread spacing, can increase resistance to vertical stresses [108]. A few studies from the review period used zirconia or PEEK implant materials to investigate the relationship between implant shape and bone density. However, there was some advice that the diameter of the zirconia implant should exceed 3.25 mm to sufficiently occupy the deep thread depth. Additionally, the cylindrical form design for the zirconia implant should be low-density.

The summary of implant geometries associated with the bone densities in each implant material is displayed in Table 6.

The overall success of dental implants also depends on other factors, including the type of implant, prosthetic connection, and the surgical technique.

First, the implant types can be categorized into one-piece and two-piece implants. Microgaps between the implant fixture and the abutment in two-piece dental implants have been associated with microleakage and bacterial contamination [110]. According to the in silico study [111], in comparison to one-piece dental implants, there were higher stress values found in the crestal bone surrounding two-piece implants. These results are used to explain the higher levels of peri-implant marginal bone loss in two-piece dental implants compared to one-piece implants.

Second, many studies have compared external and internal connection types of implants. A retrospective study compared survival rate and peri-implant marginal bone loss between different types of connections. Marginal bone loss was higher in the area around the implants with an external abutment connection after the 1-year follow-up. After five years, there was no apparent distinction between the groups with internal and external connections [112]. A systematic review compared the platform-switching and non-platform-switching implants in terms of crestal bone loss. This result confirmed that platform-switched implants are useful in clinical settings and can help to prevent bone loss around the implants [113].

Thirdly, the surgical technique plays a critical role in achieving primary stability when placing dental implants in bone that is primarily composed of medullary tissue (D3-D4). There are various surgical techniques suggested to enhance the primary stability in the low-density bone, such as undersized drilling, osteotome technique, piezoelectric bone surgery, and magnetodynamic surgery. Several studies have proved that osseodensification, underpreparation, or expander techniques improved the primary stability of low-density bone, while conventional drilling obtained lower ISQ values [2,3]. A systematic review found that while piezoelectric inserts may speed up the bone healing process, they do not affect the primary mechanical stability [114]. Recently, several authors have explored the application of magnetodynamic technology in implant rehabilitation. An in vitro study assessed the initial stability of two surgical techniques in low-density bone. Compared to the conventional method, magnetodynamic surgery seems to provide higher IT, ISQ, and RT values [115].

**Table 5:** Functional properties of implant materials.

Properties		Elastic Modulus (GPa)	Flexural/Tensile Strength (MPa)	Yield Strength (MPa)	Fracture Strength (N)	Surface Roughness	Thermal Conductivity	Hydrophobicity	Ref.
Ti	cpTi (IV)	104.1	680	485	-	More than Zr and PEEK	Yes	No	[116,117]
	TiAl_6_V_4_	110	954	729	-				[118]
	Ti-Zr	96	953	-	-				[116,119]
	Ti-24Nb-4Zr-8Sn (Ti2448)	45	850	-	-				[120]
Zr	3Y	210	900–1200	-	516–607	Less	Low	No	[121]
	ATZ	220	1800–2400	-	1064–1734				[121]
PEEK	PEEK	3–4	80	-	-	Less	Low	Yes	[121,122]
	CFR-PEEK	18	120	-	-				[121,122]
	GFR-PEEK	12	-	-	-				[96,121]

**Table 6:** Implant geometries related to the bone densities [22,47,102].

Titanium Implant		Macrogeometry			Microgeometry (Thread Design)			
Bone Density	Implant Placement Condition	Shape	Diameter	Length	Shape	Pitch	Width	Depth
High	-	- Cylindrical - Conical - Hybrid design (apical conical and crestal cylindrical)	All	All (≥6 mm)	- V-thread - Square thread - Buttress thread	Standard (1.2 mm)	Standard (0.18–0.30 mm)	Standard (0.42 mm)
Low	-	- Conical - Conical with crestal back taper - Cylindrical design with apical taper	Wider (≥4 mm)	Longer (9–11 mm)	- Reverse buttress - Self-tapping	🡇 Lower than the standard	Standard	🡅 Higher than the standard
-	Immediate	- Conical	≥4 mm	≥11 mm	- Self-tapping	-	-	-

##### Fracture Resistance

ISO 14801:2016 [123] describes a method for evaluating fracture resistance appropriate for in vitro testing. Several studies have been conducted to assess the fracture resistance behaviors (including fracture load and survival probability) of dental implants.

Implant-supported restoration using a titanium abutment and metal–ceramic crown, which has a high success rate of about 95%, is one of the traditional treatment options of choice [124]. Due to its outstanding esthetics, biocompatibility, and mechanical properties, zirconia is a considerably suitable material for constructing implant abutments or crowns in the anterior region [125]. Several studies have investigated the fracture resistance of zirconia as an abutment and values above the occlusal forces applied in the anterior section were achieved. This finding is consistent with Agustin-Panadero et al. [126], who compared the fracture resistance and failure mode of esthetic crown materials (zirconia, lithium disilicate, nanoceramic resin) and confirmed that all zirconia abutments with the three different crown materials, can withstand the physiological loads that occur in the anterior region. However, zirconia abutments bearing zirconia crowns exhibited the lowest fracture resistance (459.63 ± 66.52 N), and the most common fracture locations were the screw (80%) and abutment (15%). Focusing on the titanium implant connection interface, the interconnection between the same material outperformed zirconia abutments in fit and experienced less wear [127].

The literature has evaluated zirconia implants for their fracture resistance based on various criteria, including material selection, design, manufacturing, and restoration. Kohal et al. compared fracture strength between one-piece ATZ and Y-TZP ceramic implants [128]. The results showed that ATZ has higher mechanical stability than Y-TZP and can withstand functional loads for an anticipated 20 years. Hanes and colleagues [129] investigated the fracture resistance characteristics of titanium–zirconium, one-piece zirconia, and two-piece zirconia implants restored with zirconia crowns. The results showed that titanium–zirconium implants with prefabricated titanium abutments and zirconia crowns had higher peak fracture loads (942 ± 241 N) and better survival probability behavior than the one-piece and two-piece zirconia implants. Moreover, one-piece zirconia implants with zirconia crowns and two-piece zirconia implants with screw-retained zirconia crowns on prefabricated titanium abutment had comparable peak fracture loads (645 ± 165 N vs. 650 ± 124 N) and survival probability behavior.

A systematic meta-analysis investigated the effects of different treatments (dynamic loading, hydrothermal aging) and implant attributes (material, design, or manufacturing procedures) on the fracture resistance of zirconia implants. Regarding material, the AZT implant was more fracture-resistant than the Y-TZP implant using a one-piece design. Nonetheless, there was no statistically significant difference between the two-piece designs of ATZ and Y-TZP implants. The multi-material complex of the available two-piece zirconia implants may have caused different aging degradation behaviors under treatments (hydrothermal aging or dynamic loading). Finally, the implant components may fracture after static loading [130]. In the instance of two-piece zirconia implants combined with three distinct abutment screw materials (gold, titanium, and PEEK), there were no significant differences in fracture load among materials. The PEEK screw, however, showed inferior results. As previously stated, the evidence implies that a two-piece ATZ implant system assembled with a PEEK abutment screw is not inferior to a two-piece titanium implant in terms of its fracture resistance [131].

For implants, a one-piece design was found to be more fracture-resistant than a two-piece design [130]. Although one-piece zirconia overcame two-piece zirconia in terms of fracture resistance and insusceptibility to low-temperature degradation due to the lack of potential stress concentration at the interface and screw attachment, the lack of flexibility for detailed adjustments resulted in clinical problems for the surgeon and prosthodontist; for example, it represents a potential limitation when trying to compensate for mal-positioned implants [21]. The two-piece implant system has recently played an outstanding role on the market, but there is limited long-term clinical data and fracture analyses. The clinical fracture analysis of one-piece and two-piece zirconia implants was evaluated by Zhang et al. [21]. They found that the fracture initiation site of one-piece implants originated in the constriction area between two threads in the endosseous component. For two-piece implants, three distinct fracture initiation sites were observed at the abutment neck, internal abutment–implant connections or implant–screw connection (inner threads), and the implant body in the endosseous part. According to the investigation, the abutment neck of one-piece zirconia implants is one of the areas of stress concentration. In contrast, for two-piece zirconia implants, the quality of the abutment surface seems critically important.

The macro-design of zirconia implants is an essential parameter to consider when deciding on a system. Zirconia implants with a diameter less than or equal to 3.25 mm, a profound thread depth, and a sharp or pointed thread design are not clinically advisable due to local stress concentration [132].

Other factors affecting the fracture resistance of zirconia have been investigated, in which no statistically significant difference was found in the fracture resistance due to manufacturing method, use of different implant diameters (3.8–4.5 mm), and treatment by thermal aging or dynamic loading [130].

#### 3.2.3. Biological Properties of Dental Implant Materials to Bone Density

Biological reactions at the implant surface and bone contact are critical to the longevity of implant osseointegration and the function of the prostheses. The titanium surface interacts with water molecules and mineral ions during surgery. The surface polarity shifts and the plasma protein (albumin) binds to the surface. After that, the plasma proteins placed by the extracellular matrix protein (vitronectin) aid in cell adhesion. Cells attach to the titanium surface by binding to the vitronectin coating and other extracellular matrix proteins. After one week of implant insertion, the first bone, known as the “woven bone”, contacts the implant surface. The woven bone is then replaced by lamellar bone via the process of bone remodeling. This process may continue for years, depending on the stress distribution surrounding the implant and the bone [133,134].

Titanium is a well-known biomaterial for dental implants since it is inert and does not stimulate foreign body responses. The biomaterial serves as a scaffold for bone growth. The biomaterial’s macro- and microporosity allows osteogenic cells and blood vessels to invade, proliferate, and differentiate inside the biomaterial particles. Several surface modification techniques have been developed to improve the surface biocompatibility and bone response surrounding the implant, allowing for faster osseointegration and early loading [135].

Zirconia implants have been investigated in recent years. Zirconia is a chemically inert biomaterial with minimal local or systemic adverse effects, good cell adhesion, great tissue response, and biocompatibility with the nearby bone and soft tissue [132]. Numerous studies have shown that zirconia implants have an osteoconductive characteristic after implantation and do not have any cytotoxic effects on the bone or fibroblast [71]. Koch et al. [136] investigated the osseointegration of one-piece zirconia and titanium implants. After four months, there were no significant differences in bone level in the crestal aspect concerning material type. With the same surface modification and roughness, zirconia implants could achieve near BIC rates comparable to titanium implants. In terms of bacterial adhesion, the presence of Gram-negative anaerobic bacteria is closely linked with peri-mucositis and peri-implantitis. According to an in vitro study that evaluated the growth of biofilms on various implant materials, zirconia implants had either a comparable level or significantly less bacterial colonization than titanium implants [137]. The recommended materials for the fabrication of implant-supported prostheses are zirconia and titanium.

PEEK is a potential alternative material that has been utilized in dental implantology due to some negative aspects of titanium, including esthetic expectations, hypersensitivity reactions, and stress-shielding phenomena [96]. Even though this material has excellent biocompatibility and no cytotoxicity, it performs poorly osseointegration than titanium due to lower BIC area, less osteoblast differentiation, and less osteoconductive [96,136]. Increases in the hydrophilicity and roughness of PEEK materials can be generated via nanoscale surface treatments to overcome these drawbacks.

The surface characteristics of materials (Table 5), such as hydrophilicity, surface roughness, and surface modification [138], affect the rate and quality of osseointegration. For instance, there is better osseointegration on hydrophilic surfaces than on hydrophobic surfaces. The biological properties of various implant materials are shown in Table 7.

An increasing number of surface modifications are being introduced despite the majority of studies comparing machined surfaces with new rough surfaces [140]. Shalabi and coworkers [141] found positive correlations between surface roughness, bone-to-implant contact, and pushout strength. Surface roughness enhances osseointegration, stimulating bone formation and preventing bone resorption. Albrektsson and Wennerberg [142] classified the roughness of implant surfaces into four groups: smooth surfaces (Sa value of <0.5 µm), minimally rough surfaces (Sa value of 0.5–1 µm), moderately rough surfaces (Sa value of 1–2 µm), and rough surfaces (Sa value of >2 µm). There is currently consensus for titanium implants that a moderately rough surface with a Sa of 1 to 2 µm has the highest osseointegration potential [140]. However, this dogma may not apply to zirconia implants [143]. Subtractive and additive methods, influencing different orientations and roughness, are frequently employed to change the topography of surfaces, as shown in Table 8. Subtraction requires eliminating particles from the implant’s surface to leave pits or pores. Meanwhile, the additive approach involves adding material to the implant surface to provide a bumpy texture [140].

There are many techniques for modifying the topography of titanium implant surfaces, including mechanical, chemical, electrochemical, and layer addition [144]. Blasting and polishing are the mechanical processes most frequently used to alter a metallic surface [145]. Numerous studies have shown that the rate of osseointegration improves after treatment [146,147]. In addition, a more recent method called UV light functionalization produces micro- and nanostructured surface roughness in only the inner part of the thread, allowing for bone formation. This approach facilitates hastened osseointegration of titanium into bone [148]. Zirconia implant surfaces are treated similarly to titanium surfaces. According to clinical observations, acid etching after sandblasting resulted in the highest implant survival rate and the least mean bone loss. Sinter, slurry-modified, and sandblasted surfaces are the second and third most positive results, respectively. However, sandblasting followed by an acid etching technique produces an equivalent result to the sinter and slurry techniques regarding bone-to-implant contact. Therefore, one-piece zirconia with a sandblasted and etched surface treatment currently has the highest survival rates [143]. Several methods can be used to enhance the roughness and surface energy of PEEK materials, such as HA coating, UV light treatments, and TiO_2_ coating [138]. However, most studies on osseointegration and in vitro investigations on PEEK implants have considered only short-term consequences.

An overview of titanium, zirconia, and PEEK as dental implant materials in clinical settings is shown in Table 9.

### 3.3. Clinical Application of Dental Implants and Their Survival Rates

#### 3.3.1. Survival Rate of Alternative Implant Materials

A significant factor determining the long-term success rate of implants is peri-implant mean boss loss. According to the guidelines of the consensus report of the First European Workshop on Periodontology, successful results are achieved when bone decreases of less than 1.5 mm are noted during the first year of functional loading and 0.2 mm annual bone loss is observed [149]. Many studies have reported the success rate of dental implant materials in clinical settings. Long-term usage of titanium implants has demonstrated the material’s excellent success rate in various applications [150], including single/partial implant-supported restorations, removable implant-retained/supported overdenture, and fixed implant-supported prostheses. In the past decade, zirconia implants have been employed as an alternative to titanium implants when patients have metal allergies or esthetic concerns. Zirconia has equivalent biological, physical, and biocompatibility properties to titanium [17]. In addition to material selection, several manufacturers have tried to modify the surface topography using subtractive and additive techniques to improve their properties [143]. Meta-analyses of clinical trials on alternative implant materials and success rates are summarized in Table 10.

The findings of two systematic reviews reported the overall survival rate of zirconia implants after 1 to 7.8 years of observation. Adanez and colleagues [152] found that zirconia implants had a 95% survival rate (76% to 100%) after 1 to 7 years of surveillance. In terms of implant design, one-piece designs had a survival rate of 95% (95% Cl 91–97%) following 1 to 7 years of monitoring. The two-piece designs had an overall survival rate of 94% (95% CI 87–97%) after observation periods of between 1 and 3 years. They also observed a failure rate of 6.44% for one-piece designs and 13.66% for two-piece designs. After one year, the mean MBL of zirconia implants was 0.89 mm (95% CI 0.60–1.18). According to Borges et al. [151], the overall mean MBL was 0.80 mm (95% CI: 0.60 to 1.00) and 1.01 (95% CI: 0.72 to 1.29) after 1- and 2-year observation periods, consecutively. The survival rate ranged from 71.2% at one year to 100% at 7.8 years. In conclusion, zirconia implants, particularly one-piece designs, demonstrated acceptable survival and marginal bone loss values in short-term (1-year) monitoring periods, consistent with the standard consensus [149]. However, due to a lack of clinical support, they did not suggest using two-piece zirconia in clinical settings.

Bone augmentation or narrow-diameter dental implants (NDIs) are alternative options for treatment when there is not enough bone for placing regular-diameter implants. Nevertheless, patients with parafunctional habits or areas with a high occlusal load have serious concerns about the resistance and strength of NDIs [153]. A Ti-Zr alloy was created (Roxolid; Institute Straumann AG, Basel, Switzerland) to enhance mechanical strength and biocompatibility [154]. Altuna et al. [155] concluded that narrow-diameter Ti-Zr dental implants had survival and success rates (>95%) and marginal bone loss (<1 mm) equivalent to those of regular-diameter titanium implants in the short term.

#### 3.3.2. Survival Rate of Dental Implants Related to Bone Density

Bone density is a significant variable for anticipating stress–strain distribution at the peri-implant area, influencing bone modeling and remodeling and, consequently, the success or failure rates of dental implants. Furthermore, the difference in stiffness between implant material and peri-implant bone causes marginal bone loss and aseptic implant loosening due to stress-shielding phenomena.

Concerning bone density, mean bone loss after implant placement determines the clinical success rate of dental implant materials. Our review focuses on the factors, such as material types, properties, and bone densities (according to Misch’s classification [35]), that influence clinical outcome parameters, as indicated in Table 11. Unclear or undefined bone quality is not included in this table.

The seven included studies selected for analysis were published between 2012 and 2022 and had data regarding bone qualities according to Misch’s classification. Only one study (n = 18) evaluated zirconia material in environments of low bone density. Other studies (n = 398) examined the effects of titanium under different bone conditions. The evaluated studies had follow-up periods varying between three and twelve months.

The level of peri-implant bone loss is measured based on periapical films using the parallel technique. Regardless of the material and follow-up period, low bone quality (D_3/4_) tended to have more MBL than high bone quality (D_1/2_) [156,157,158,159,160,161]. For instance, the mean MBL reduction reported by Held et al. [119], at roughly 1.46 mm, is close to the borderline value recommendation (1.50 mm). However, the data suggested that the hydrophilic implants have good osseointegration characteristics even in low-quality bone. This finding is consistent with a previous systematic analysis, which found that the survival rates of dental implants depending on the bone density were type I, 97.6%; type II, 96.2%; type III, 96.5%; and type IV, 88.8%. Additionally, compared to machined surface implants, treated surface implants had a greater survival rate (97.1%) when placed in low-density bone. This higher survival rate may explain the surface treatment’s role in facilitating tighter cell–titanium interactions, which enhance the bone tissue’s biological and biomechanical effects [162].

**Table 11:** Clinical parameters of dental materials related to bone density in the included studies.

Study/Year	Study Type	Material	Implant	Surface	Site	Geometry	Mean MBL (Mean ± SD)	Follow-Up (Month)
Aldebes et al. 2022 [163]	RCTs	One-piece zirconia (Y-TZP)	18	Sandblasting surface	Premolar	Anatomic zirconia implant	D_3/4_: 0.61 ± 0.23	12
Makary et al. 2019 [156]	CT		16				D_1_: 0.20 ± 0.27	12
		Titanium	23	Ca^2+^ on the SLA-treated surface	All	Different diameters of 4, 4.5, 5, 5.5 mm with 10 mm length	D_2/3_: 0.11 ± 0.11	
			7				D_4_: 0.33 ± 0.14	
Hingsammer et al. 2017 [157]	CT		15				D_1_: 0.66 ± 0.72	12
		Titanium	38	TiUnite surface and machine neck	Posterior	Short implants	D_2/3:_ 0.60 ± 0.77	
			21				D_4_: 0.65 ± 0.68	
De Santis et al. 2016 [158]	CT	Titanium	35	TiUnite surface	All	Tapered and double-variable thread design, apical drilling blades	D_2/3:_ 0.68 ± 0.65	6
			109				D_4_: 0.73 ± 0.46	
Cannizzaro et al. 2012 [159]	RCTs	Titanium	68	Dual etched and covered with nanoscale calcium phosphate crystal	Immediate loading site	4 mm diameter with tapered-with external connection	D_123_: 0.26 ± 0.35	6
Rossi et al. 2014 [160]	CT	Titanium	31	SLActive surface with moderate rough	Posterior	6 mm length with 4.1 and 4.8 mm diameter	D_123:_ 0.55 ± 0.80	12
Held et al. 2013 [161]	CT	Titanium	35	Sandblasted and acid-etched surface	All	ELEMENT RC implants with 4, 4.5, 5 mm diameter and 8, 9.5, 11.5, 12, 14 mm length	D_3/4_: 1.46 ± 0.75	3

Aldebes et al. [163] observed that the mean MBL of one-piece modified anatomic zirconia implants was 0.61 mm in the low bone type. The findings also indicated that the one-piece design and zirconia implant material, considered tissue-friendly and biocompatible, may offset lower MBL. As a result, changes in the peri-implant bone density or the level of bone-to-implant contact are indicators of the implants’ osseointegration abilities. Makaray et al. [156] did show, however, that IT values might be regulated by matching implant geometry to bone type. High IT values did not impact marginal bone levels when using a specific implant design.

## 4. Conclusions

- Implant material, implant design, and surgical techniques are pivotal factors affecting the success rates of dental implant placement in low-density bone.
- Both titanium and zirconia implants are widely accepted materials in the market. Nonetheless, PEEK implants serve as an alternative material for specific cases, such as those involving poor bone conditions, bruxism, and esthetic concerns.
- Modified implant topography, strengthened implant geometry, and a suitable surgical technique are selected and used to achieve high survival and success rates and attain superior clinical results.
- In low-density conditions,
  o Titanium provides a better chance of achieving initial stability due to the best mechanical performance among the three materials.
  o Conical titanium implant design, wider diameter, longer length, reverse buttress with self-tapping, small thread pitch, and deep thread depth are recommended.
  o Surgical techniques, such as underpreparation, osteotome technique, and magnetodynamic surgery, play a critical role in achieving primary stability. However, piezoelectric surgery does not affect the initial stability but does affect the secondary stability.
- Regardless of the material and follow-up period, low bone quality tended to have more marginal bone loss than high bone quality.
- Further study is required to identify an optimal implant material in terms of the bone state in clinical settings.

## Figures and Tables

**Table 1:** Search strategy according to three databases (PubMed, Scopus, Embase).

Databases	Search Strategy
PubMed/Medline	MeSH terms: (Dental Implants) AND ((Osseointegration) OR (bone–implant interface) OR (survival rate)) Text words: ((Titanium) OR (titanium implant) OR (zirconia) OR (zirconium oxide) OR (yttria-stabilized tetragonal zirconia) OR (zirconia implant) OR (ceramic implant) OR (PEEK) OR (Polyetheretherketone)) AND ((bone density) OR (bone mineral) OR (bone condition)) AND ((success rate) OR (marginal bone loss) OR (bone–implant contact) OR (removal torque) OR (osteoblasts) OR (cell proliferation) OR (bone remodeling))
Scopus	(Dental implants) AND ((titanium) OR (titanium implant) OR (zirconia) OR (zirconium oxide) OR (yttria-stabilized tetragonal zirconia) OR (zirconia implant) OR (ceramic implant) OR (PEEK) OR (Polyetheretherketone)) AND ((bone density) OR (bone mineral) OR (bone condition)) AND ((osseointegration) OR (bone–implant-interface) OR (survival rate) OR (success rate) OR (marginal bone loss) OR (bone–implant contact) OR (removal torque) OR (osteoblasts) OR (cell proliferation) OR (bone remodeling))
Embase	(Dental implants) AND ((titanium) OR (titanium implant) OR (zirconia) OR (zirconium oxide) OR (yttria-stabilized tetragonal zirconia) OR (zirconia implant) OR (ceramic implant) OR (PEEK) OR (Polyetheretherketone)) AND ((bone density) OR (bone mineral) OR (bone condition)) AND ((osseointegration) OR (bone–implant-interface) OR (survival rate) OR (success rate) OR (marginal bone loss) OR (bone–implant contact) OR (removal torque) OR (osteoblasts) OR (cell proliferation) OR (bone remodeling))

**Table 2:** Percentage of different bone densities (bone type) in different jaw areas [29].

Type	Anterior Maxilla (%)	Posterior Maxilla (%)	Anterior Mandible (%)	Posterior Mandible (%)
D_1_	0	0	6	3
D_2_	25	10	66	50
D_3_	65	50	25	46
D_4_	10	40	3	1

**Table 3:** Hounsfield values for different substances.

Substance		Hounsfield Units
Air		−1000
Water		0
Bone	Cancellous	+300 to +400 [37]
	Cortical	+500 to +1900 [37,38,39]
	Anterior maxilla	+600 to +700 [34,40]
	Posterior maxilla	+300 to +400 [34,40]
	Anterior mandible	+800 to +1100 [34,40]
	Posterior mandible	+500 to +600 [34,40]

**Table 4:** Elastic modulus of alveolar bone and implant materials [47,49].

Substances	Elastic Modulus (GPa)
Zirconia Oxide	210
TiAl_6_V_4_	110
CpTi (Grade IV)	104.1
PEEK	3.6
Carbon-reinforced PEEK	18
Cortical	15.85 ± 2.10
Trabecular	7.95 ± 2.10

**Table 7:** Biological properties of implant materials.

Properties	Titanium	Zirconia	PEEK	Citation
Esthetics	Acceptable	Superior to Ti	Superior to Ti	[117,121]
Tissue adhesion	Acceptable	Superior to Ti	Lesser or similar to Ti and Zr	[117,121,136,139]
Biocompatibility	Acceptable	Superior to Ti	Superior to Ti	[117,121,139]
Bacterial formation	Moderate	Lesser or similar to Ti	Least	[117,121,139]
Cytotoxicity	Moderate	Lower than Ti	Least	[121]
Osseointegration	High	Similar to Ti (after surface treatment)	Lesser than Ti and Zr	[96,121,136]

**Table 8:** Techniques frequently used to change the topography of implant surfaces [140,143].

Titanium		Zirconia	
Subtractive	Additive	Subtractive	Additive
Electropolishing	HA and other calcium phosphate coatings	Sandblasting	Additive sintering and slurrying
Mechanical polishing	TPS surfaces	Sandblasting followed by acid etching	Injection molding
Blasting	Ion deposition	Laser ablation	Additionally heat-treated
Etching			
Oxidation			
UV light			

**Table 9:** Comparative information about the clinical application of dental implant materials containing titanium, zirconia, and PEEK.

Clinical Settings	Titanium	Zirconia	PEEK
Bone density			
- High-density	Yes	Yes	Maybe
- Low-density	Yes	No (low osseointegration)	Questionable (yes, mechanical aspect) (no, biological aspect)
Occlusal force	Normal to high	Normal	High (bruxism)
Esthetic benefits	Maybe	Yes	Yes
Allergy	Maybe	Low	Low

**Table 10:** Meta-analyses of clinical studies on alternative implant materials and survival rates.

Study/Year	Material	Marginal Bone Loss	Survival Rate (%)
Borges et al. 2020 [151]	Zirconia	0.80 mm (95% CI: 0.60 to 1.00) at a 1-year post-loading.	71.2% at 1 year to 100% at 7.8 years.
		1.01 mm (95% CI: 0.72 to 1.29) at a 2-year post-loading	
Adanez et al. 2018 [152]	Zirconia	0.89 mm (95% CI: 0.60 to 1.18) at a 1-year post-loading period.	76% to 100% after observation periods between 1 and 7 years.
			The mean survival rate was 95% after an observation period between 1 and 7 years (one-piece zirconia: 95%, two-piece zirconia: 94%).
Borges et al. 2020 [151]	Titanium–zirconia alloy	0.36 ± 0.06 mm after 1 year.	98.4% at 1 year after implant placement.
		0.41 ± 0.09 mm after 2 years.	97.7% at 2 years after implant placement.

## Data Availability

Not applicable.

## Lists of Abbreviations

Abbreviations	Explanation
TiO_2_	Titanium oxide
3Y-TZP	3 mol% yttria–tetragonal zirconia polycrystal
ATZ	Alumina-toughened zirconia
BIC	Bone–implant interface contact
LTD	Low-temperature degradation
HUs	Hounsfield units
CT	Computer tomography
CBCT	Cone beam computed tomography
GVs	Gray values
PEEK	Polyetheretherketone
IT	Insertion torque
RFA	Resonance frequency analysis
ISQ	Implant stability quotient
RTQ	Removal torque
SLM	Selective laser fusion
EBM	Electron beam fusion
ZrO_2_	Zirconium dioxide
PSZ	Partially stabilized zirconia
TZP	Tetragonal zirconia polycrystal
CFR-PEEK	Carbon fiber reinforcement polyetheretherketone
GFR-PEEK	Glass fiber reinforcement polyetheretherketone
GPa	Gigapascal
Ti_6_Al_4_V	Titanium–6 Aluminum–4 Vanadium
CpTi	Commercially pure titanium
HA	Hydroxyapatite
TPS	Titanium plasma-sprayed
UV	Ultraviolet
RCTs	Randomized Clinical Trials
Non-RCT	Non-Randomized Clinical Trial
